# Accuracy of intercellular adhesion molecule-1 for diagnosing sepsis: A systematic review and meta-analysis protocol

**DOI:** 10.1097/MD.0000000000016019

**Published:** 2019-06-14

**Authors:** Xiang-jin Li, En-li Tan, Cheng-peng Zhao, Juan Yan

**Affiliations:** a Department of Pediatric; b Department of Geriatric Respiratory; c Department of Oncology, the First Hospital of Lanzhou University; d Clinical College of Traditonal Chinese Medicine, Gansu University of Traditional Chinese Medicine, Lanzhou, China.

**Keywords:** diagnosis, ICAM-1, meta-analysis, protocol, sepsis, systematic review

## Abstract

**Background:**

Sepsis is a complex and life-threatening systemic disease. A positive blood culture is the criterion standard of diagnosis for sepsis; however, it does not produce results for 24 to 72 hours. Besides, the clinical manifestations of sepsis are variable and nonspecific. Therefore, a new diagnostic biomarker for diagnosis of sepsis should be developed. The present study aims to assess the diagnostic value of intercellular adhesion molecule-1 (ICAM-1) in individuals with sepsis.

**Methods:**

The literature will be searched in PubMed, EMBASE, the Cochrane Library, and Web of Science databases from the inception of each database up to June 2019. The methodological quality of eligible study will be assessed by Quality Assessment of Diagnostic Accuracy Studies tool-2 (QUADAS-2). Stata 15.1 software (version 15.1, Stata Corporation) will be used to calculate the pooled sensitivity, pooled specificity, pooled positive likelihood ratio, pooled negative likelihood ratio, pooled diagnostic odds ratio, pre-test probability, post-test probability, and summary receiver-operating characteristic curve for diagnostic value of ICAM-1. The *I*^2^ statistic will be used to test heterogeneity. Subgroup analysis will be used to explore the source of inconsistency. GRADE (Grading of Recommendations Assessment, Development, and Evaluation) system will be used to assess the certainty of evidence. This study will be conducted fully following the Preferred Reporting Items for Systematic Reviews and Meta-Analyses of diagnostic test accuracy.

**Results and conclusions:**

Our study will detect the potential of ICAM-1 for diagnosing the patients with sepsis and the results will be submitted to a peer-reviewed journal.

**Discussion:**

The evidence will indicate that ICAM-1 is a valuable biomarker for detecting sepsis. This is a protocol of systematic review and meta-analysis, so the ethical approval and patient consent are not required.

## 1 Introduction

Sepsis is a complex and life-threatening systemic disease, with various of etiological factors and wide spectrum of severity.^[1,2]^ It has emerged as a major global health and economic burden, especially severe sepsis and septic shock.^[3–5]^ Although the treatment has been improved quickly, severe sepsis affects 700,000 residents, remains a high mortality of 30% to 50% and costs 20 million dollars each year in the United Stated.^[6–9]^ Currently, a positive blood culture, as reference standard, exists for proof of sepsis. However, the blood cultures do not produce results for 24 to 72 hours, and cultures are less sensitive because of previous antibiotic treatment.^[2,10]^ Additionally, early clinical signs of sepsis are variable and nonspecific, including sleepiness, anxiety, and weight loss.^[2,11]^ Therefore, considering these limitations in diagnosis of sepsis and the importance of reducing mortality and cost of care,^[12–14]^ a new and reliable biomarker that diagnose sepsis should be developed.

It is well known that endothelial activation is crucial in sepsis.^[4,15]^ Intercellular adhesion molecule-1 (ICAM-1), mediating adherence between cells and extracellular matrix, could release in cascade to damage the vascular endothelial cells during sepsis.^[16–18]^ ICAM-1 has been investigated in sepsis. To date, many studies showed that its level is increased in patients with sepsis.^[19–21]^ Bavunoglu et al found that ICAM-1 had a high sensitivity (99%) and specificity (99%) for detecting sepsis.^[21]^ Thus, ICAM-1 might be an effective biomarker for diagnosing sepsis.

The diagnostic value of ICAM-1 in sepsis has been reported in several studies; however, these results are variable. Therefore, our study aims to conduct a systematic review and meta-analysis to indicate the potential value of ICAM-1 for diagnosing sepsis.

## 2 Methods

### 2.1 Protocol and registration

This study will be conducted fully following the Preferred Reporting Items for Systematic Reviews and Meta-Analyses of diagnostic test accuracy criteria.^[22]^ Besides, the study protocol has been registered on the international prospective register of systematic review (PROSPERO) (Registration number: CRD42019127217).

### 2.2 Data sources

PubMed, EMBASE, the Cochrane Library, and Web of Science databases will be used to search relevant citations from their inception up to June 2019. Our search terms will include “sepsis,” “septicemia,” “blood poisoning,” “intercellular adhesion molecule-1”, and “ICAM-1”. A comprehensive literature search strategy which bases on the following combination of MeSH terms and all fields will be used for PubMed database: (((“Sepsis”[Mesh]) OR (((((((sepsis) OR severe sepsis) OR pyemia) OR pyohemia) OR pyaemia) OR septicemia) OR blood poisoning))) AND ((“Intercellular Adhesion Molecule-1”[Mesh]) OR (((((intercellular adhesion molecule-1) OR ICAM-1) OR soluble intercellular adhesion molecule-1) OR sICAM-1) OR CD54)). Additionally, we will manually search the major references of the included studies and relevant reviews to track any potential studies.

### 2.3 Eligibility criteria

#### 2.3.1 Study design

We will include the cohort, cross-sectional, and case–control studies.

#### 2.3.2 Participants

Reporting on individuals with sepsis will be included. There will be no restrictions on publication year, study regions, and sexes.

#### 2.3.3 Index test

We will include only studies of ICAM-1 for the detection of sepsis and provision the blood as the index test.

#### 2.3.4 Reference standard

The gold standard for discriminate patients with sepsis is microbiological culture, and/or clinical and radiological tests.

#### 2.3.5 Outcomes

Studies reported sensitivity and specificity of ICAM-1 for detecting sepsis, or reported sufficient information which can calculate sensitivity and specificity.

#### 2.3.6 Exclusion criterion

We will exclude studies with <5 patients. Reviews, animal experiments, case reports, conference abstracts without full texts, and duplication of the same trials will also be excluded.

### 2.4 Literature selection

Initial search records will be imported into the EndNote version 8. After the duplicated records are deleted, 2 reviewers will screen independently the title and abstract of studies according to the eligibility criteria. Then, the full-texts of all potentially relevant studies will be downloaded and further confirm the eligibility. Any disagreements will be resolved by discussing.

### 2.5 Data extraction

Data extraction http://dict.youdao.com/w/eng/extraction_of_data/-keyfrom=dict.phrase.wordgroup from the selected articles will be independently performed by two reviewers. Any disagreements will be resolved by discussing and reaching in accordance. The data of interest will include: author, publication date, study regions, year of enrollment, study design, number of participants (sepsis individuals and health controls), the age of participants, index test (measurement method and cut-off value), severity of sepsis; and sensitivity, specificity, true-positive (individuals with positive ICAM-1 who have proven sepsis), false-positive (individuals with positive ICAM-1 who do not have proven sepsis), false-negative (individuals with negative ICAM-1 who have proven sepsis), true-negative (individuals with negative ICAM-1 who do not have proven sepsis) of ICAM-1 for diagnosing patients with sepsis.

### 2.6 Quality assessment

Two reviewers will independently assess the methodological quality of included studies using the Quality Assessment of Diagnostic Accuracy Studies tool-2 (QUADAS-2).^[23]^ Bias (whether the selection criteria are clearly described) will be related to patient selection. Bias (whether sufficient details of ICAM-1 test have been included) will be related to the index test. Bias (whether a reference standard of the selected samples is included and is used for all patients, whether there is enough time between the reference test and ICAM-1 test) will be related to the reference standard. Bias (whether the test results reported are interpretable) will be related to the flow and timing. For unresolved disagreements, a third reviewer is consulted. RevMan 5.3 software will be used to draw the graph of methodological quality.

### 2.7 Statistical analysis

#### 2.7.1 Data analysis

Stata 15.1 software (Stata Corporation) will be used to perform the statistical analysis and draw funnel plots. Spearman correlation analysis will be used to assess the threshold effect and *P* < .05 indicates a significant threshold effect. Heterogeneity will be evaluated by Cochrane Q test and *I*^2^ statistic.^[24]^*I*^2^ could be calculated from the formula of *I*^2^ = 100% × (Q – df)/Q. If *I*^2^ is <50% or the *P* value is >.1, a fixed-effect model will be used for pooling the data, whereas, if *I*^2^ is >50% or the *P* value is <.1, then there is more heterogeneity among studies, and a bivariate random-effects model will be utilized for pooling the data. We will calculate the pooled sensitivity, pooled specificity, pooled positive likelihood ratio, pooled negative likelihood ratio, pooled diagnostic odds ratio, pre-test probability, and post-test probability.^[25]^ On the basis of the sensitivity and specificity, we will further construct the summary receiver-operating characteristic curve and calculate the area under the curve to evaluate the detecting accuracy of ICAM-1.^[26]^

#### 2.7.2 Subgroup analysis

We will conduct subgroup analysis and explore the possible sources of heterogeneity, including the age of participants, the methods of ICAM-1, and the cut-off of ICAM-1.

#### 2.7.3 Assessment of reporting bias

Stata 15.1 software will be used to analyze the publication bias. The Deek funnel plot asymmetry test is assessed for publication bias, when we include >10 studies.^[27]^

### 2.8 Certainty of evidence assessment

The certainty (quality) of evidence will be rated by using the guidelines of the Grading of Recommendations, Assessment, Development, and Evaluation (GRADE) system, and using 4 levels: high certainty, moderate certainty, low certainty, and very low certainty.^[28]^

### 2.9 Ethics and dissemination

All analyses will be done according to previous published studies, so there are no patient consent, ethical approval, and institutional review board required.

## 3 Discussion

Sepsis is still a death-related disease in the worldwide.^[2]^ Because the early symptoms and physical signs of sepsis vary, it is easy to misdiagnose it as pneumonia. In clinical practice, although microbiological examinations can be used to make an accurate diagnosis of sepsis, they have a low sensitivity and are time-consuming and ineffective. Previous studies have shown that ICAM-1 can promptly be detected and may be a promising marker to diagnose sepsis.^[19–21]^ However, to date, these comparable studies have not been evaluated with a systematic approach. We will conduct the meta-analysis to evaluate the potential value of ICAM-1 for detecting the sepsis. Our study will provide the evidence of ICAM-1 in diagnosing sepsis and will be valuable for clinical decision.

## Author contributions

LXJ planned and designed the research; LXJ, TEL, ZCP and YJ tested the feasibility of the study; LXJ wrote the manuscript; all authors approved the final version of the manuscript.

**Conceptualization:** Xiang-jin Li, En-li Tan, Cheng-peng Zhao, Juan Yan.

**Investigation:** Xiang-jin Li, En-li Tan, Cheng-peng Zhao, Juan Yan.

**Methodology:** Xiang-jin Li, En-li Tan, Cheng-peng Zhao, Juan Yan.

**Project administration:** Xiang-jin Li, Cheng-peng Zhao, Juan Yan.

**Software:** Xiang-jin Li.

**Writing – original draft:** Xiang-jin Li.

**Writing – review & editing:** Xiang-jin Li, En-li Tan, Cheng-peng Zhao, Juan Yan.
